# Effect of intermittent normobaric hypoxia on executive functions assessed with different cognitive tests in healthy young subjects

**DOI:** 10.3389/fpsyg.2025.1688199

**Published:** 2025-12-03

**Authors:** Cristina Rochel-Vera, Francisco Esparza-Ros, Raquel Vaquero-Cristóbal, Antonio Martinez-Nicolas

**Affiliations:** 1 Research Group of Physical Exercise and Human Performance, Mare Nostrum Campus, University of Murcia, Murcia, Spain; 2 Injury Prevention in Sport Research Group (PRELEDE), UCAM Universidad Católica de Murcia, Murcia, Spain; 3 Research Group Movement Sciences and Sport (MS&SPORT), Department of Physical Activity and Sport, Faculty of Sport Sciences, University of Murcia, Murcia, Spain; 4 Department of Physiology, Human Physiology Area, Sports Sciences Faculty, University of Murcia, Murcia, Spain; 5 Chronobiology Lab, Department of Physiology, College of Biology, Universidad de Murcia, Mare Nostrum Campus, IUIE, IMIB-Arrixaca, Murcia, Spain; 6 Centro de Investigación Biomédica en Red de Fragilidad y Envejecimiento Saludable (CIBERFES), Madrid, Spain

**Keywords:** cognitive tests, intermittent hypoxia, executive functions, stress, acute effect

## Abstract

**Objective:**

The main objective of this study was to analyze the effects of an intermittent normobaric hypoxia session on executive functions assessed with different cognitive tests given to healthy young subjects.

**Methods:**

In this randomized trial with a between-subject design, 27 healthy volunteers were divided into an experimental group (EG; *n* = 13) and a control group (CG; *n* = 14). Participants completed tasks assessing deductive reasoning, response inhibition, and visuospatial working memory. The experimental group performed these tasks before and after an intermittent hypoxia session (12%, 4,400 m) with the iAltitude simulator, while the control group completed them before and after normoxic conditions without hypoxia exposure.

**Results:**

Both groups showed an increase in the score obtained in the Double Trouble test (*p* = 0.001–0.002; 95%CI = −19.11, −4.23; *η*^2^ = 0.552–0.660), the CG showed also improvement in the level in the Odd One Out test (*p* = 0.034; 95%CI = 3.69, −0.17; *η*^2^ = 0.301). However, the Group (IH vs. Control) × Time (Pre vs. Post) interaction was not significant for any of the executive function variables, indicating similar patterns of change across groups both in the overall sample and when analyses were conducted separately by sex. Significant differences were found in SaO₂ (*p* = 0.001; 95%CI = 2.89, 10.18) and HR (*p* = 0.012; 95%CI = −15.55, −1.37) after hypoxia in the EG.

**Conclusion:**

A single session of intermittent hypoxia did not produce clear changes in executive function against control group, which suggests that it may not alter cognitive function at the acute level.

## 1 Introduction

Intermittent hypoxia (IH) is based on the alternation in breathing of hypoxic air and normoxic air, resulting in changes in blood oxygenation, as found during exposure to high altitude (Gangwar et al., 2019; Timon et al., 2023). The initial responses to hypoxia are compensatory, activating mechanisms such as increased sympathetic nervous system activity and blood pressure, ensuring adequate oxygenated blood supply to vital organs (Twomey et al., 2017). These physiological responses maintain homeostasis during hypoxia (Koehler et al., 2018).

Intermittent hypoxia has emerged as a promising strategy to modulate sympathetic activation without incurring the adverse effects associated with prolonged hypoxia exposure (Navarrete-Opazo and Mitchell, 2014). Scientific evidence suggests that brief and repeated exposures typical of IH may induce transient sympathetic activation, promoting beneficial cardiovascular and metabolic adaptations, such as improvements in baroreflex sensitivity and tissue perfusion (Raberin et al., 2023). Unlike sustained hypoxia, which is associated with endothelial dysfunction and chronic hypertension, IH appears to stimulate adaptive mechanisms without compromising homeostasis, typically with hypoxia at 9–16% FiO₂, with different program lengths (from 1 day to 10 weeks), cycles (from 1 to 3) and exposure time (from 5 min to 12 h) (Navarrete-Opazo and Mitchell, 2014). Moreover, recent studies suggest that IH may have neuroprotective effects and enhance cognitive performance by boosting synaptic plasticity, neurogenesis, and resistance to oxidative stress in the brain, with protocols such as 8 cycles of 5 min hypoxia at 10% FiO₂ alternating with 5 min normoxia (room air), 3 times per week for 8 weeks (Manukhina et al., 2016; Wang et al., 2020). These findings suggest that IH could be an effective tool for improving performance in both clinical and sports settings, offering a novel avenue for further research.

IH protocols and hypoxia simulation devices have experienced a rapid increase in popularity in the medical field (Törpel et al., 2019). In fact, IH training protocols at approximately 13.5% FiO₂ are widely used in sports medicine to improve the aerobic capacity of athletes by increasing erythrocyte mass (Törpel et al., 2019). In the clinical setting, IH has been used as a non-pharmacological strategy to treat a wide range of pathophysiological conditions, such as obesity (Timon et al., 2023), hypertension (Glazachev et al., 2017), chronic lung disease (Timon et al., 2023), bronchial asthma (Janssen Daalen et al., 2022), coronary heart disease (Glazachev et al., 2017), diabetes mellitus (Janssen Daalen et al., 2022), neurological diseases such as Parkinson’s disease (Basovich, 2010), and in psychological disorders (Coppel et al., 2015), among others (Janssen Daalen et al., 2022), at FiO₂ levels ranging from 12 to 16%. It has been suggested that IH at FiO₂ levels ranging from 13 to 16% may have positive effects on the aforementioned diseases (Timon et al., 2023), but current data are contradictory, as IH-induced physiological and metabolic changes and adaptations vary depending on the duration, the FiO₂ and time of hypoxic exposure, as well as other factors such as age or repeated exposure to hypoxia training (Törpel et al., 2020; Prabhakar et al., 2022).

The role of IH in cognitive and executive functions is currently being studied (Pun et al., 2019). The brain, which is highly sensitive to hypoxia, relies on a constant oxygen supply for its energy demands, as neurons have a reduced capacity to store energy (McMorris et al., 2017). It has been proven that exposure to chronic hypoxia affects executive brain functions (Pun et al., 2018), such as attention (Davranche et al., 2016; Ochi et al., 2018), information processing (Neuhaus and Hinkelbein, 2014; Behrens, 2022) and memory (McMorris et al., 2017), with adverse effects on reaction time and error rate during cognitive tasks (McMorris et al., 2017). On the other hand, several studies have examined the acute effects of hypoxia on cognitive performance. Systematic reviews and meta-analyses have shown that acute exposure to hypoxia from 11 to 21% of FiO_2_ can impair reaction time, memory, and executive function, although the magnitude of these effects depends on factors such as the severity and duration of hypoxia and the type of cognitive task used (Limmer and Platen, 2018; Post et al., 2023; Ramírez-de la Cruz et al., 2025). Among the different cognitive domains, complex executive functions and memory appear particularly vulnerable, especially at lower oxygen saturations (Post et al., 2023).

In human studies, acute normobaric hypoxia at FiO₂ levels ranging from 12 to 21% was found to decrease cognitive performance (such as executive function and reaction time) regardless of sex, suggesting that short-term exposure affects cognition similarly in men and women (Karayigit et al., 2022a,b). However, some studies focused exclusively on one gender, such as young men or women, and did not make direct comparisons between sexes (Chroboczek et al., 2021; Lei et al., 2019).

Despite this substantial body of research, important gaps remain. Many prior studies have not systematically addressed how individual factors such as sex differences modulate cognitive responses to acute hypoxia, nor have they consistently used standardized, domain-specific cognitive tests that can detect subtle changes in executive function, working memory, and reasoning (Karayigit et al., 2022a,b). Additionally, there is considerable heterogeneity in hypoxia protocols (e.g., normobaric vs. hypobaric, intermittent vs. continuous), the age and health status of the participants, and the type of cognitive task the subjects must perform (Turner et al., 2015; Pun et al., 2019).

However, IH does not necessarily lead to cognitive impairment, and controlled exposure may activate the sympathetic system without compromising cognitive function despite exposing participants to 4 cycles of 10 min at 13% of FiO_2_, followed by 5 min of normoxia (Zhang Q. et al., 2024). In fact, IH could trigger the release of growth factors and other neuroprotective substances, supporting brain cell maintenance and repair (Dale et al., 2014). Other studies suggest that IH exposure about 10% of FiO_2_ may enhance cognitive functions such as attention and alertness, although its impact on memory and other executive functions requires further investigation (Xing et al., 2014; Boulares et al., 2024).

Intermittent normobaric hypoxia at approximately 10% of FiO₂ has been suggested to enhance neuroplasticity, improve cognitive function, and contribute to a better quality of life, potentially helping to prevent or treat cognitive decline (Virués-Ortega et al., 2004; Schega et al., 2013; Xu et al., 2014; Wang et al., 2020). Despite these promising findings, research in this area remains limited, and results are still inconclusive. It appears that the effects of normobaric intermittent hypoxia on cognition may depend on factors such as exposure dose and duration (Pun et al., 2019). Furthermore, few studies have specifically examined its effects in healthy subjects, revealing an important gap in current research (Chroboczek et al., 2022).

The inconsistency of findings in the current literature limits the clinical and practical applicability of IH protocols (Yuan et al., 2022). Resolving these contradictions is crucial for the development of safe and effective interventions in areas such as neurorehabilitation, cognitive enhancement in vulnerable populations, and the preparation of individuals regularly exposed to hypoxic environments (e.g., athletes, pilots, and high-altitude workers) (Aragón-Vela et al., 2020; Yuan et al., 2022). Clarifying the specific effects of IH on executive functions may therefore contribute to optimizing its therapeutic and preventive applications (Yuan et al., 2022; Baillieul et al., 2017).

Delving into executive functions, different parameters can be evaluated within this factor. Response inhibition and sustained attention, measured by the Double Trouble test, is essential for focusing and resisting interference (Nichols et al., 2021a,b); visuospatial working memory, assessed by the Monkey Ladder test, supports navigation and manipulation of visual information (Wild C. J. et al., 2022); and deductive reasoning, evaluated with Odd One Out test, is fundamental for logical problem-solving (Honarmand et al., 2019; Sujanthan et al., 2025; Nichols et al., 2021a,b). These functions have relevance in daily cognitive demands and their known sensitivity to alterations in oxygen availability (Post et al., 2023).

Thus, the aim of the current study was to analyze the effects of an intermittent normobaric hypoxia session on executive functions, assessed with different cognitive tests, in healthy young adults, and analyze the influence of sex on these effects. It was hypothesized that a single session of intermittent normobaric hypoxia might not impair executive functions in healthy young individuals and that these effects would not differ between sexes.

## 2 Materials and methods

### 2.1 Design

A randomized controlled trial was conducted to analyze the acute effects of an intermittent session at a simulated altitude of 4,400 m above sea level with an oxygen saturation of 12% on executive tasks and physiological variables. Measurements were taken before (pre-test) and after (post-test) the session. The tests were performed under medical supervision. The entire process was carried out at the laboratory of the Physical Exercise and Human Performance Research Group of the University of Murcia (Spain). The clinical trial registration number is NCT07027410 (24/05/2025).

The trial design was registered and followed the Consolidated Standards of Reporting Trials guidelines (CONSORT) (Schulz et al., 2010). This study was approved by the Research Ethics Committee of University of Murcia (code: 4145/2022), and the entire study was conducted following the precepts of the Declaration of Helsinki. The participants signed the informed consent form and were able to leave the study at any time.

### 2.2 Participants

The sample size was calculated by using the Rstudio statistical software (v. 3.15.0; Rstudio Inc., Boston, MA, United States) and setting the statistical significance at *α* = 0.05. The Standard Deviation (SD) was obtained from previous research on response inhibition hypoxia effects (SD = 3.9) (Komiyama et al., 2015). This technique for sample size calculation is based on the use of the SD, a constant, and the estimated effect size. This methodology for sample size calculation has been used in previous research (Kadam and Bhalerao, 2010). With an estimated error (*d*) of 2.12 for the response inhibition score, a significance level of *α* = 0.05, and a power of 95% (1 − *β* = 0.95), the minimum sample needed was 13 participants.

The inclusion criteria were: (1) adults aged between 18 and 40 years old. The exclusion criteria were: (1) pregnancy; (2) prior experience in hypoxia training; (3) previous experience in executive function tests; (4) personal or family history of cardiovascular diseases or chronic conditions; (5) diagnosed cardiac disorders.

Participants were recruited via social media platforms through targeted advertisements and study invitations. The recruitment posts provided a brief overview of the study objectives, inclusion criteria, and a contact link for those interested in participating. Participants were also recruited by snowballing through other subjects. Recruitment took place in March 2023. Individuals who expressed interest were then screened to ensure they met the inclusion criteria. Once eligibility was confirmed, they were invited to participate in the study.

Finally, 27 adults aged 18–40 years (mean age = 24.2 ± 4.8 years) participated in the present study. Among them, 13 participants were randomly assigned to the experimental group (mean age = 26.5 ± 5.7 years) and 14 to the control group (mean age = 21.9 ± 2.2 years).

**Figure 1:**
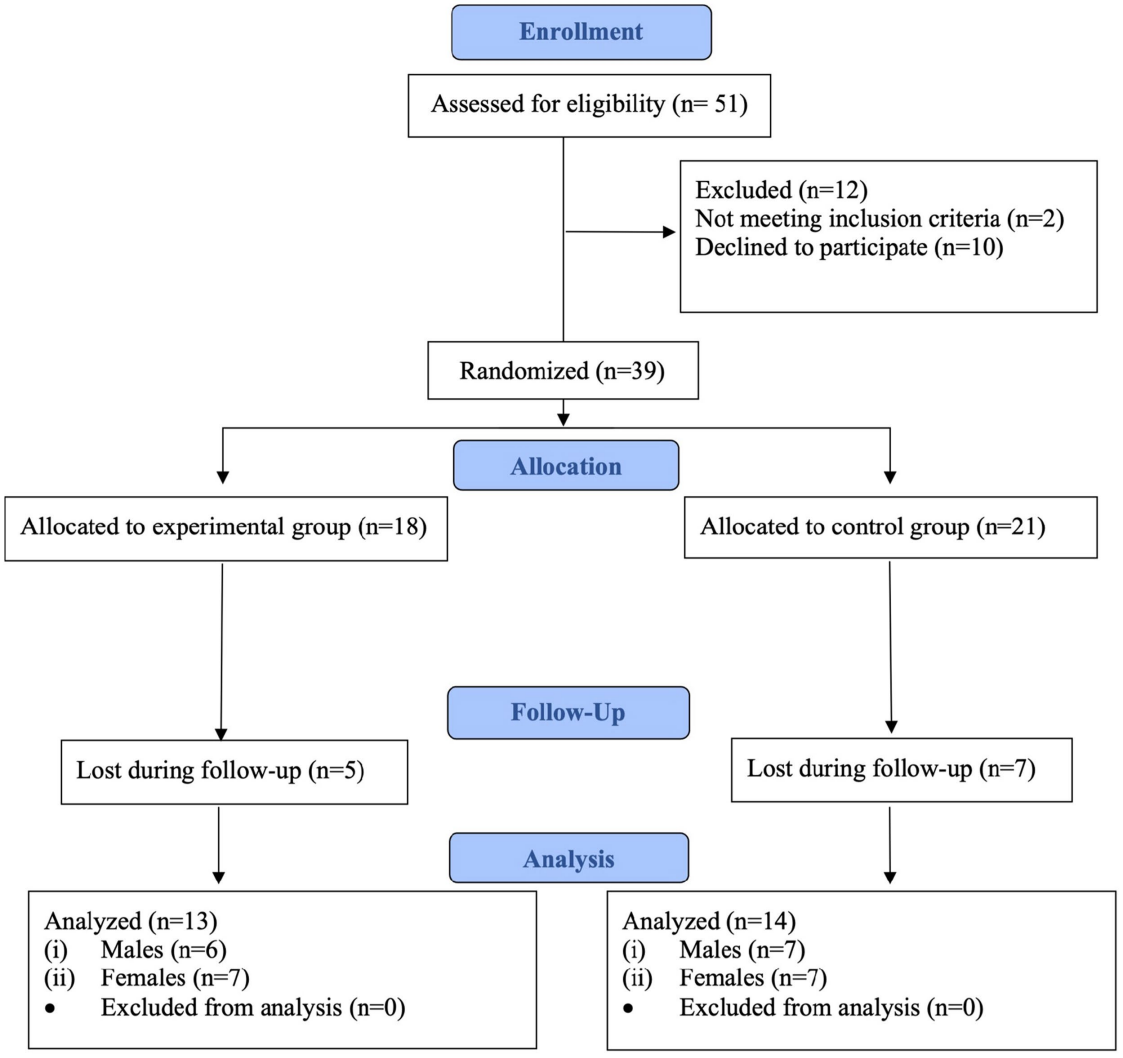
Flow diagram of the sample selection.

Flow diagram of the sample selection is showed in Figure 1.

### 2.3 Randomization and blinding

The principal investigator performed the stratification randomization using a computer-generated random number table, with an allocation ratio 1:1, with the presence of other investigators not involved in the study. Participants were randomly assigned to either the experimental group (EG) or the control group (CG) based on sex. The randomization process initially allocated 18 participants to the EG and 21 to the CG. After accounting for losses during follow-up, data from 13 participants in the EG (6 males, 7 females) and 14 participants in the CG (7 males, 7 females) were analyzed.

Pre-test measurements were taken after the randomization. The researchers involved in the measurements were blinded to group assignments. In the post-test, the researchers were blinded to the participant’s group and pre-test measurements. The researchers conducting the hypoxia training for the EG were also blinded to pre-test measurements.

### 2.4 Instruments

For the collection of sociodemographic data and previous experience regarding age, pregnancy status, previous experience in hypoxia training, previous experience in performing executive tests, and personal and/or family history of cardiovascular pathology and other possible chronic diseases, a questionnaire designed *ad hoc* based on the one used in previous research (Albertus-Cámara et al., 2023) was used. For the detection of cardiac pathologies, an electrocardiogram was performed with a Cardioline Click^®^ electrocardiograph (Cardioline S.p. A., Trento, Italy).

Executive functions were assessed in the current study through three validated cognitive tests: the response inhibition was evaluated with the Double Trouble Test (Hurt et al., 2025), the visuospatial working memory was evaluated with the Monkey Ladder Test (Hurt et al., 2025), and deductive reasoning was evaluated with the Odd One Out Test (Honarmand et al., 2019; Sujanthan et al., 2025; Nichols et al., 2021a,b). These tests were chosen from the Cambridge Brain Sciences platform for their reliability and sensitivity to cognitive changes (Hosseini et al., 2023; Wild C. et al., 2022; Wild C. J. et al., 2022), for to be good indicators of cognitive function (Honarmand et al., 2019; Sternin et al., 2019; Sujanthan et al., 2025; Nichols et al., 2021a,b) and for they are widely used in previous research (Hurt et al., 2025; Riganello et al., 2023; Wild C. et al., 2022). In fact, although traditional clinical tests could have been used, the current design opted for digital platform-based cognitive performance tests, as they are standardized and have been validated in previous studies (Anguera et al., 2013).

These tests were completed by EG and CG participants at two different measurement times (pre-test and post-test). In addition, EG participants underwent a series of physiological parameter tests including systolic blood pressure (SBP), diastolic blood pressure (DBP), arterial oxygen saturation (SaO_2_), and heart rate (HR) before and after hypoxia training, following the protocol from previous research (Albertus-Cámara et al., 2023).

#### 2.4.1 Assessment of executive functions

The assessment of executive functions included three tests: the response inhibition was evaluated with the Double Trouble Test (Hurt et al., 2025), the visuospatial working memory was evaluated with the Monkey Ladder Test (Hurt et al., 2025), and deductive reasoning was evaluated with the Odd One Out Test (Honarmand et al., 2019; Sujanthan et al., 2025; Nichols et al., 2021a,b). These tests are part of a cognitive training platform called ‘Cambridge Brain Sciences’, which is a platform for cognitive training in both research and professional use for the measurement of cognitive functions (Honarmand et al., 2019), widely used in previous research (Hurt et al., 2025; Riganello et al., 2023; Wild C. J. et al., 2022). All tasks were performed using a MacBook Air^®^ (Apple, California, United States), and participants responded via button press on the laptop.

The Double Trouble test evaluates response inhibition, verbal ability and attention. In this task, three color words are presented on the screen (one at the top and two at the bottom) each displayed in incongruent ink colors. Participants must select the word at the bottom that describes the color of the word at the top by pressing a button on the laptop. For example, if the top word reads “green” but is written in red, the correct response is to select the word “red” among the two options at the bottom. This task is a more complex version of the classic Stroop task, requiring not only color naming but also distinguishing between two potential answers, which adds an additional cognitive step of information reassignment between representations (Nichols et al., 2021a,b). Participants were given 90 s to complete as many trials as possible, earning +1 point for each correct response and −1 point for each incorrect response. No inter-trial interval was applied. The final score corresponds to the total number of correct responses, minus incorrect answers, after 90 s.

The Monkey Ladder test assesses visuospatial working memory. On each trial, several boxes appear in random positions on the screen, each containing a number in sequential order (e.g., 1 to 9). The numbers remain visible for 3 s before disappearing. Participants must then touch the boxes in ascending numerical order. Task difficulty is adaptive: the number of boxes increases after a correct response and decreases after an incorrect one. The test includes up to 25 levels and ends after three consecutive errors. There is no time limit per trial, and the final score corresponds to the highest sequence length (number of boxes) correctly recalled by each participant (Wild C. J. et al., 2022).

The Odd One Out test evaluates working memory, deductive reasoning and shifting. In this task, participants are shown nine figures and must identify the one that does not belong to the group, based on shape, color, or relational properties. Each trial remains on screen until the participant responds, with no inter-trial delay. Task difficulty is adaptive: after a correct response, the test increases complexity by presenting more subtle or intricate patterns, whereas after an incorrect response, it decreases difficulty with simpler and more distinguishable figures. The test includes up to 20 levels and lasts 180 s, during which participants must provide as many correct answers as possible. The score is calculated as the number of correct responses minus the number of errors. The test ends when the time limit expires. Both the highest level achieved and the final score obtained by each participant were recorded (Hynes et al., 2014; Owen et al., 2010).

#### 2.4.2 Assessment of physiological parameters

A SBP and DBP assessment was performed on the EG participants using a Beurer^®^ upper arm blood pressure monitor (Beurer GmbH, BM26, Germany) to measure blood pressure (Albertus-Cámara et al., 2023). Furthermore, SaO_2_ and HR were monitored using the altitude simulator iAltitude^®^ Trainer v2.7 (iAltitude, Madrid, Spain) (Albertus-Cámara et al., 2023).

### 2.5 Procedure

After the recruitment and selection of the participants, meetings were held to explain the study’s purpose and randomization. The tests were carried out in the laboratory of the Physical Exercise and Human Performance Research Group of the University of Murcia (Spain) in a room whose atmospheric conditions were standardized and maintained throughout the data collection process, with temperature controlled in 24 °C and relative humidity of 50%, as these conditions are commonly used in cognitive and physical performance research to ensure participant comfort and reliable results (Donnan et al., 2021).

**Figure 2:**
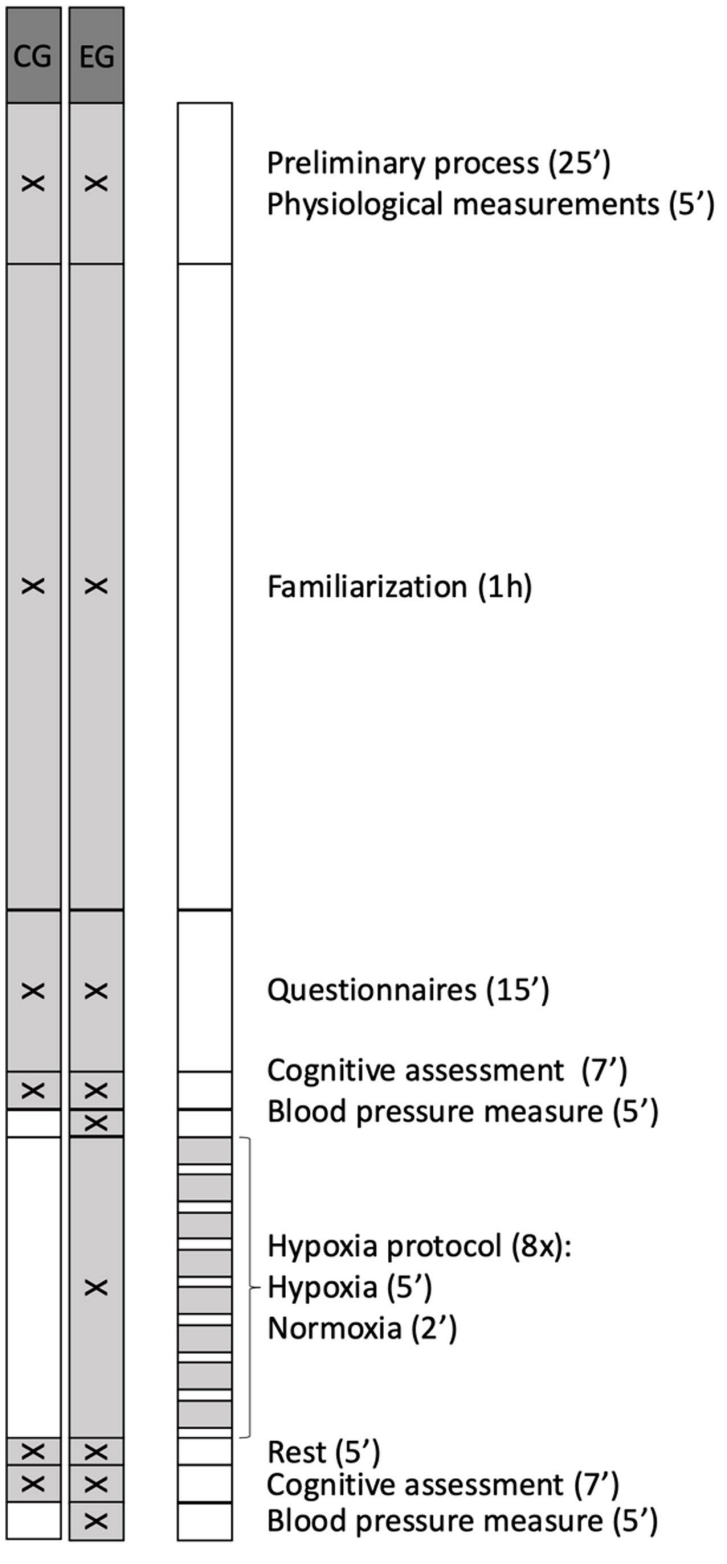
Outline of the phases of the investigation. EG, experimental group; CG, control group.

Outline of the phases of the investigation is showed in Figure 2. A physician with more than 30 years of experience made the diagnosis of cardiac alterations by means of an electrocardiogram performed with a Cardioline Click^®^ electrocardiograph (Cardioline S.p. A., Trento, Italy). If no cardiac pathologies were found, the procedure continued.

A familiarization protocol was carried out 1 h before starting the measurements, ensuring that participants understood the dynamics of the executive tests, as well as the hypoxia protocol in the case of the EG participants, thus avoiding the enhancement of executive test performance by repetition of the task during the all-data phase. To avoid cognitive fatigue and to ensure that participants reached the experimental phase in optimal conditions, clear and standardized instructions on each cognitive test were provided prior to the start of the test, ensuring correct understanding and execution of the tasks. Furthermore, task familiarization and regular break, and pre-session instructions were included, as these measures have been shown to help maintain performance and well-being during prolonged cognitive tasks following previous research (Nichols et al., 2021a,b; Guo et al., 2023; Medina-Inojosa et al., 2024). More specifically, familiarization protocol consisted of randomly performing two non-consecutive attempts at each of the three tests (Monkey Ladder, Odd One Out and Double Trouble) in the same conditions previous described, in line with previous studies that typically use between 1 and 3 familiarization attempts before formal data collection (Goldberg et al., 2015). A three-minute break was included between tests. In addition, EG participants were given an explanation on how to use and put on and take off your mask when the machine instructs you to do so; and were instructed to breathe normally while wearing it, but no prior familiarization with the hypoxia protocol was carried out. The total duration of this familiarization session was 1 h.

To begin data collection, all the participants self-completed the sociodemographic and previous experience questionnaire. After this, the participants performed the Double Trouble Test, Monkey Ladder Test, and Odd One Out Test in a randomized order (pre-test) (Metzler-Baddeley et al., 2016), using a MacBook Air^®^ (Apple, California, United States) as the assessment device and the Cambridge Brain Science platform to perform the tests.

After that, the participants in the EG remained seated at rest for 5 min and had their blood pressure measured and EG participants underwent an IH session (FiO_2_ = 12%, equivalent to 4,400 m) using the iAltitude® Trainer v2.7 altitude simulator (iAltitude, Madrid, Spain) (Albertus-Cámara et al., 2023). Participants had to sit in an armchair, and while facing a screen displaying the simulator instructions, they had to put on or take off the mask when the screen indicated them to alternate cycles of hypoxia with cycles of normoxia. During the intervallic training session, which lasted 1 h, eight cycles were exchanged, each consisting of two phases: a long hypoxia phase (5 min) and a short normoxia phase (2 min).

The values of SaO₂ and HR were recorded using the iAltitude^®^ simulator (iAltitude, Madrid, Spain), which measured these parameters at the beginning and at the end of the IH session. After the IH session blood pressure was measured again. Meanwhile, the participants in the CG remained seated at rest in normoxic conditions for the same amount of time as the blood pressure assessment and the IH protocol. At the end of this phase, both EG and CG participants repeated the executive tests (post-test).

### 2.6 Data analysis

Statistical tests were selected based on the study design, following previous references (Heinemann, 2003). Normality of the variables was analyzed using the Shapiro–Wilk test, and kurtosis and skewness analysis. The Levene’s test was used to assess the homogeneity between the EG and CG in the study variables. As the data followed a normal distribution, parametric tests were used for analysis. Mean and standard deviation (M ± SD) were used as descriptive values for the sample. A mixed model ANOVA was carried out to analyze intragroup differences in the Double Trouble test score, Monkey Ladder test level and score, and Odd One Out test level and score for general sample, in men and women. Subsequently, an analysis of covariance (ANCOVA) was carried out to analyze the differences in the study variables when sex was included. For the analysis of change, an ANOVA was performed to compare the difference between the pre and post EG results with respect to the difference between the pre and post CG results for general sample, in men and women. Two ANCOVAs with general sample were subsequently performed with the covariate sex. Partial eta squared (*η*^2^) was used to calculate the effect size and was defined as small: ES ≥ 0.10; moderate: ES ≥ 0.30; large: ES ≥ 1.2; or very large: ES ≥ 2.0, with an error of *p* < 0.05 (Hopkins et al., 2009). A value of *p* < 0.05 was set to determine statistical significance. The statistical analysis was performed with the SPSS statistical package (version 28.0; SPSS Inc.).

## 3 Results

Table 1 shows the differences between the pre-test and post-test scores in the EG and the CG in the Double Trouble test (score), Monkey Ladder test (level and score), and Odd One Out test (level and score). The participants in the EG showed an increase in the score obtained in the double trouble test after having been subjected to a hypoxia situation (*p* = 0.002; 95%CI = −15.31, −4.23; *η*^2^ = 0.552), without there having been a change in the performance of the rest of the executive tasks after the intervention (*p* > 0.05). On the other hand, the CG showed an improvement in the score obtained in the double trouble test (*p* = 0.001; 95%CI = −19.11, −7.61; *η*^2^ = 0.660), as well as in the level reached in the Odd One Out test (*p* = 0.034; 95%CI = 3.69, −0.17; *η*^2^ = 0.301), without significant differences in the rest of the tests (*p* > 0.05).

**Table 1:** Differences between the pre- and post-intervention measurements in the experimental group (EG) and control group (CG) in executive tasks in general sample.

Measurements							
Group	Pre-test	Post-test	Pre-post diff.	*F*	*p*	95% CI	*η* ^2^
Double trouble test (score)							
EG	20.4 ± 14.5	30.2 ± 15.2	−9.8 ± 2.5	14.77	0.002	−15.31, −4.23	0.552
CG	22.4 ± 15.8	35.7 ± 15.5	−13.4 ± 2.6	25.23	0.001	−19.11, −7.61	0.660
Monkey Ladder test (level)							
EG	6.8 ± 1.1	6.5 ± 1.5	0.4 ± 0.4	0.85	0.374	−0.52, 1.29	0.065
CG	7.3 ± 1.1	7.0 ± 1.5	0.3 ± 0.3	1.15	0.302	−0.28, 0.86	0.082
Monkey Ladder test (score)							
EG	7.5 ± 0.8	7.5 ± 0.9	−0.1 ± 0.4	0.04	0.837	−0.87, 0.72	0.004
CG	7.8 ± 1.1	7.7 ± 1.2	0.1 ± 0.3	0.05	0.818	−0.58, 0.73	0.004
Odd One Out test (level)							
EG	14.5 ± 3.2	15.4 ± 2.2	−0.9 ± 0.8	1.18	0.299	−2.77, 0.93	0.089
CG	15.1 ± 2.6	17.1 ± 1.7	−1.9 ± 0.8	5.59	0.034	−3.69, −0.17	0.301
Odd One Out test (score)							
EG	10.7 ± 3.9	10.2 ± 2.7	0.5 ± 1.1	0.22	0.646	−1.95, 3.03	0.018
CG	11.1 ± 4.5	11.1 ± 4.7	0.1 ± 1.5	0.01	1.000	−3.44, 3.44	0.001

Diff., differences; EG, experimental group; CG, control group.

Table 2 shows the differences between the pre- and post-intervention measurements in the EG and CG in executive tasks among men. The CG showed a significant improvement in the score of the Double Trouble test after the intervention (*p* = 0.005; 95%CI = −19.22, −3.92; *η*^2^ = 0.299), while no significant differences were found in the rest of the executive tasks (*p* > 0.05).

**Table 2:** Differences between the pre- and post-intervention measurements in the experimental group (EG) and control group (CG) in executive tasks in men.

Measurements							
Group	Pre-test	Post-test	Pre-post diff.	*F*	*p*	95% CI	*η* ^2^
Double Trouble test (score)							
EG	14.3 ± 13.6	22.0 ± 15.8	−7.7 ± 3.9	3.68	0.067	−15.93, 0.59	0.138
CG	26.4 ± 17.9	38.0 ± 21.3	−11.6 ± 3.7	9.79	0.005	−19.22, −3.92	0.299
Monkey Ladder test (level)							
EG	7.2 ± 0.9	6.7 ± 1.5	0.5 ± 0.5	0.89	0.355	−0.59, 1.59	0.037
CG	7.3 ± 1.4	6.7 ± 2.1	0.6 ± 0.5	1.36	0.255	−0.44, 1.58	0.056
Monkey Ladder test (score)							
EG	7.7 ± 1.0	7.7 ± 0.8	0.0 ± 0.5	0.00	1.000	−1.07, 1.07	0.000
CG	7.8 ± 1.6	7.6 ± 1.5	0.3 ± 0.5	0.35	0.558	−0.70, 1.28	0.015
Odd One Out test (level)							
EG	14.8 ± 2.2	13.8 ± 1.2	1.0 ± 1.2	0.73	0.402	−1.42, 3.42	0.031
CG	14.4 ± 3.2	15.8 ± 1.4	−1.4 ± 1.1	1.74	0.200	−3.67, 0.81	0.070
Odd One Out test (score)							
EG	12.5 ± 2.7	9.0 ± 2.4	3.5 ± 1.9	3.32	0.082	−0.47, 7.47	0.126
CG	11.0 ± 3.6	9.0 ± 5.9	2.0 ± 1.8	1.26	0.272	−1.68, 5.68	0.052

Diff., differences; EG, experimental group; CG, control group.

Table 3 presents the differences between the pre- and post-intervention measurements in the EG and CG in executive tasks among women. The EG showed a significant increase in the score of the Double Trouble test (*p* = 0.005; 95%CI = −19.22, −3.92; *η*^2^ = 0.299) and in the level reached in the Odd One Out test (*p* = 0.026; 95%CI = −4.81, −0.33; *η*^2^ = 0.197). Similarly, the CG exhibited significant improvements in the score in the Double Trouble test (*p* = 0.000; 95%CI = −22.79, −7.49; *η*^2^ = 0.442) and in the Odd One Out test level (*p* = 0.035; 95%CI = −4.67, −0.18; *η*^2^ = 0.179). No significant differences were observed in the remaining executive tasks (*p* > 0.05).

**Table 3:** Differences between the pre- and post-intervention measurements in the experimental group (EG) and control group (CG) in executive tasks in women.

Measurements							
Group	Pre-test	Post-test	Pre-post diff.	*F*	*p*	95% CI	*η* ^2^
Double Trouble test (score)							
EG	25.6 ± 14.9	37.1 ± 11.3	−11.6 ± 3.7	9.792	0.005	−19.22, −3.92	0.299
CG	18.3 ± 13.7	33.4 ± 7.3	−15.1 ± 3.7	16.769	0.000	−22.79, −7.49	0.442
Monkey Ladder test (level)							
EG	6.6 ± 1.3	6.3 ± 1.6	0.3 ± 0.5	0.34	0.565	−0.73, 1.29	0.015
CG	7.3 ± 0.7	7.3 ± 0.7	0.0 ± 0.5	0.00	1.000	−1.01, 1.01	0.000
Monkey Ladder test (score)							
EG	7.3 ± 7.7	7.4 ± 1.1	−0.1 ± 0.5	0.08	0.769	−1.14, 0.85	0.004
CG	7.7 ± 0.5	7.8 ± 1.1	−0.1 ± 0.5	0.08	0.769	−1.14, 0.85	0.004
Odd One Out test (level)							
EG	14.1 ± 3.9	16.7 ± 1.9	−2.6 ± 1.1	5.63	0.026	−4.81, −0.33	0.197
CG	15.8 ± 1.9	18.3 ± 1.1	−2.4 ± 1.1	5.02	0.035	−4.67, −0.18	0.179
Odd One Out test (score)							
EG	9.1 ± 4.3	11.1 ± 2.8	−2.0 ± 1.8	1.26	0.272	−5.68, 1.68	0.052
CG	11.1 ± 5.7	13.1 ± 1.7	−2.0 ± 1.8	1.26	0.272	−5.68, 1.68	0.052

Diff., differences; EG, experimental group; CG, control group.

Table 4 shows an analysis of pre-post differences found in the EG and CG, and also the effect of the inclusion of the covariate sex. No differences were found for the pre-post-test change between the EG and the CG for any of the executive variables analyzed (*p* > 0.05), nor an effect of the covariate sex (*p* > 0.05). When analyses were conducted separately by sex, no differences were found in the analysis of pre-post differences found in the EG and CG in either men or women.

**Table 4:** Differences in the pre-post-test changes between the EG and CG in general sample and stratified by sex.

Variable	Pre-Post EG – Pre-post CG	*F*	*p*	95%CI diff	*η* ^2^
Double trouble test (score)	3.6 ± 3.7	0.95	0.340	−11.19, 4.01	0.036
Monkey Ladder (level)	0.1 ± 0.5	0.04	0.841	−1.10, 0.90	0.002
Monkey Ladder (score)	0.1 ± 0.5	0.09	0.757	−0.83, 1.12	0.004
Odd One Out (level)	1.0 ± 1.2	0.73	0.401	−3.43, 1.42	0.028
Odd One Out (score)	0.5 ± 1.9	0.07	0.788	−4.62, 3.55	0.003
Men (*n* = 13)					
Double trouble test (score)	3.9 ± 4.0	0.92	0.357	−5.04, 12.85	0.077
Monkey Ladder (level)	0.1 ± 0.6	0.01	0.909	−1.41, 1.27	0.001
Monkey Ladder (score)	−0.3 ± 0.6	0.22	0.651	−1.64, 1.07	0.019
Odd One Out (level)	2.4 ± 1.7	1.91	0.194	−1.44, 6.29	0.148
Odd One Out (score)	1.5 ± 2.9	0.26	0.623	−5.04, 8.04	0.023
Women (*n* = 14)					
Double trouble test (score)	3.6 ± 6.2	0.33	0.575	−9.93, 17.08	0.027
Monkey Ladder (level)	0.3 ± 0.8	0.13	0.720	−1.41, 1.98	0.011
Monkey Ladder (score)	0.0 ± 0.7	0.00	1.000	−1.64, 1.64	0.000
Odd One Out (level)	0.1 ± 1.4	0.01	0.919	−3.14, 2.85	0.001
Odd One Out (score)	0.0 ± 2.1	0.00	1.000	−4.71, 4.71	0.000

EG, experimental group; CG, control group.

Table 5 shows the comparison of the EG’s cardiovascular parameters between the pre-test and the post-test stratified by sex. Statistically significant differences were found in the SaO_2_ (*p* = 0.001; 95% CI = 2.89, 10.18) and HR (*p* = 0.012; 95%CI = −15.55, −1.37) variables. When analyzed separately by sex, significant changes in SaO₂ were found in both men (*p* = 0.020; 95%CI = 1.63, 12.03) and women (*p* = 0.028; 95%CI = 0.99, 12.43). In addition, HR showed a significant change in women (*p* = 0.047; 95%CI = −20.6, −0.20), whereas no significant change was observed in men.

**Table 5:** Changes in the physiological parameters between the pre-test and the post-test in the EG stratified by sex.

Variable	Pre-test	Post-test	Mean ± SD of the difference between pre-test and post-test	t	p	95%CI (max; min)
General						
Systolic blood pressure (mm Hg)	118.2 ± 11.4	118.0 ± 16.9	0.2 ± 14.1	0.059	0.477	8.73, −8.27
Diastolic blood pressure (mm Hg)	75.7 ± 9.5	74.1 ± 10.5	1.5 ± 9.7	0.572	0.289	7.39, −4.32
SaO_2_ (%)	99.2 ± 1.0	92.3 ± 5.1	6.5 ± 6.0	3.906	0.001	10.18, 2.89
HR (beats per minute)	74.3 ± 9.9	82.8 ± 13.0	−8.5 ± 11.7	−2.599	0.012	−1.37, −15.55
Men						
Systolic blood pressure (mm Hg)	124.8 ± 15.6	124.3 ± 12.9	0.8 ± 20.1	0.102	0.923	−20.25, 21.92
Diastolic blood pressure (mm Hg)	71.7 ± 14.9	72.2 ± 11.9	−1.7 ± 10.5	−0.388	0.714	−12.71, 9.37
SaO_2_ (%)	99.2 ± 0.9	99.0 ± 1.3	6.8 ± 4.9	3.377	0.020	1.63, 12.03
HR (beats per minute)	72.2 ± 11.8	72.2 ± 13.5	−5.0 ± 8.4	−1.447	0.207	−13.88, 3.88
Women						
Systolic blood pressure (mm Hg)	119.3 ± 10.9	113.0 ± 7.2	−0.3 ± 7.6	−0.099	0.925	−7.36, 6.79
Diastolic blood pressure (mm Hg)	75.8 ± 9.5	78.3 ± 6.6	4.3 ± 8.7	1.296	0.243	−3.81, 12.38
SaO_2_ (%)	98.7 ± 1.9	99.3 ± 0.7	6.7 ± 6.1	2.873	0.028	0.99, 12.43
HR (beats per minute)	76.1 ± 8.5	77.1 ± 10.2	−10.4 ± 11.1	−2.495	0.047	−20.6, −0.20

SaO_2_, oxygen saturation; HR, heart rate.

## 4 Discussion

The aim of the current study was to analyze the effects of an intermittent normobaric hypoxia session on executive functions assessed with different cognitive tests given to healthy young adults, specifically targeting response inhibition, visuospatial working memory, and deductive reasoning, which are critical for daily cognitive demands such as focusing attention, manipulating visual–spatial information, and solving problems logically (Diamond, 2013; Kazali, 2025). In the current study, EG showed improvements in response inhibition following IH exposure, with a similar tendency for CG participants. No significant changes were observed in visuospatial working memory in any group for general sample and after divided by sex. Furthermore, in women, the deductive reasoning showed significant improvement in both the EG and CG, while in men no significant change was observed. However, the most notable result was that there were no differences in the evolution of the EG and CG in any of the cases, either for the general sample or when divided by sex. So, the general results of this study indicate that exposure to an IH protocol did not have significant effect in executive function, showing the EG a similar evolution to the CG.

Some studies have evaluated the effects of hypoxia induced by simulated altitude on cognitive function with contradictory results. Cognitive responses may vary depending on the simulated altitude, duration of hypoxia exposure, the phase of the test in which participants are assessed (preliminary, hypoxia, recovery), the type of hypoxia (hypobaric or normobaric), the protocol applied, the complexity of the cognitive tasks, and the individual’s familiarity with the tasks, all of which can influence the outcomes (Virués-Ortega et al., 2004). This scenario reflects the considerable heterogeneity in hypoxia studies, suggesting the need for a more systematic approach to better understand the specific effects of each hypoxia protocol on cognitive function (Williams et al., 2019; Ramírez-de la Cruz et al., 2025).

More specifically, one study has showed a cognitive decline during acute and prolonged high-altitude in real exposure at 4,350 m in young men, particularly in information processing in terms of speed and accuracy (Davranche et al., 2016). However, it has been suggested that in real-life exposure to hypoxia, cognitive performance impairment may be more related to symptoms of acute altitude sickness than to altitude-induced hypoxia (Issa et al., 2016). Other studies have shown that acute exposure to IH with hypoxia at 11.8% of FiO₂ and normoxia at a room air at 21% of FiO₂ in young participants can increase the number of commission errors during the Conners’ Continuous Performance Test (CCPT-II), although reaction time and omission errors did not differ (Uchida et al., 2020). These changes could be related to reduced oxygenation in the frontal lobe and an alteration in the connectivity of the brain network (Uchida et al., 2020). Furthermore, Williams et al. (2019) demonstrated that reductions in SaO₂ induced by 1 h of normobaric hypoxia at FiO₂ levels ranging from 12 to 20% are associated with decreased cognitive performance, such as assessing working memory measured by accuracy in the n-back task. Similarly, an acute decline in performance on the Stroop task, which assesses attention, inhibitory control, and cognitive flexibility, was observed following normobaric hypoxia at FiO₂ of 13.5% (Ochi et al., 2018).

However, some studies using moderate hypoxia or short exposures under simulated hypoxic conditions, as has been done in the current study, report minimal or no significant effects on cognitive accuracy or reaction time, suggesting that the severity and duration of hypoxia are critical factors. More specifically, it has been shown that the influence of acute normobaric hypoxia on cognitive function in young adults depends on the percentage of FiO₂, with a decrease in reaction time observed at FiO₂ = 11% (5,100 m, 16,735 ft), while no deterioration was found in the other conditions analyzed (FiO₂ = 15%, 2,750 m, FiO₂ = 13%, 3,800 m). The exposures were applied continuously during each trial using the GO_2_Altitude^®^ Hypoxicator system; however, the study did not specify the exact duration of each exposure, the number or length of recovery periods, nor the number of cycles applied (Ramírez-de la Cruz et al., 2025). Another study conducted on a young population found that exposure to hypoxia affected cognitive performance only under severe hypoxia conditions (FiO₂ = 10.5%), following a single 10-min continuous exposure separated by a 10-min recovery period (Patrick et al., 2023). These results are similar to those obtained in the current study, although the intermittent normobaric hypoxia protocol used in this study (12%, 4,400 m; eight cycles of 5 min hypoxia and 2 min normoxia, total duration 1 h) differs from that used in previous ones. In addition, most previous studies that analyzed the effect of IH in cognitive tasks lack a control group, which limits the ability to determine if an intervention truly causes observed effects, leading to biased, unreliable, and potentially invalid conclusions (Pithon, 2013; Moser, 2019). In contrast, the present study had a randomized controlled trial design, which provide the most reliable evidence for determining whether an intervention truly causes an effect, by minimizing bias and confounding (Lim and In, 2019).

Furthermore, the improvement in inhibitory response could reflect a combination of acute hypoxia-induced adaptive responses and practice effects (Ando et al., 2020). Theoretically, these executive functions could be influenced by hypoxia because the prefrontal cortex and associated neural circuits, which are critical for attention, visuospatial working memory, and deductive reasoning, are highly oxygen-dependent (Chen et al., 2023). However, controlled IH from 9 to 16% of FiO_2,_ from 3 to 15 cycles of 3 to 10 min with different duration for the intervention may also induce adaptive neuroplastic responses, potentially enhancing specific cognitive functions through hormetic mechanisms (Zhang et al., 2023). Similarly, participants underwent cycles of 10 min hypoxia – 5 min normoxia for 1 h per session, three sessions per week over 6 weeks, with FiO₂ adjusted to maintain SpO₂ levels of 90–80% across the intervention increase positive effect which increased cognitive performance and quality of life in older adults (Schega et al., 2013). Moreover, IH conditioning combined with exercise training has been shown to enhance cognitive performance in healthy older adults (Baillieul et al., 2017). Similarly, in individuals with spinal cord injury, a single IH session consisting of 15 cycles of 90-s hypoxia (FiO₂ = 9%) followed by 60-s normoxia (FiO₂ = 21%) also demonstrated acute improvements in respiratory function (Hayes et al., 2014).

Recent human studies have begun to address sex differences in cognitive and physiological responses to hypoxia, though findings remain mixed. For example, some research indicates that while females may experience lower peripheral oxygen saturation and report more headaches during hypoxic exposure, sex does not consistently predict the degree of cognitive performance decline, likely due to high individual variability and the influence of other factors such as age and body mass index (Vento et al., 2022). Other studies suggest that women may have greater resistance to hypoxia and higher baseline cerebral blood flow, possibly linked to estrogen, yet these physiological advantages do not always translate into measurable differences in cognitive outcomes under hypoxic conditions (Raberin et al., 2024; Peltonen et al., 2016). Moreover, the literature highlights the need for larger, well-controlled studies to clarify whether sex-based differences in executive function and cognitive flexibility under IH are robust and generalizable (Vento et al., 2022; Raberin et al., 2024).

An additional finding worth noting of the current study is the significant effect of sex observed in deductive reasoning and response inhibition, both in the EG and the CG. Although sex was not a primary variable of interest, its influence suggests the presence of possible sex-based differences in cognitive performance, particularly in tasks that require response inhibition and deductive reasoning after IH situations. Previous literature has reported that men and women may rely on different cognitive strategies in problem-solving and spatial reasoning tasks, potentially contributing to performance differences in this type of executive function test (Yuan et al., 2019; Chen et al., 2024). In particular, neurovascular or hormonal factors, such as estrogen levels, have been linked to variations in prefrontal cortex activity under acute stress, which may modulate performance on executive tasks (Ando et al., 2020; Galea et al., 2017; Stark et al., 2006). Additionally, learning process related to executive functions could also be influences by psychosocial or cultural factors related to gender roles could have also played a role (Grenell and Carlson, 2021). However, due to the limited sample size of the current research and the exploratory nature of this analysis, these results should be interpreted with caution. Future studies should consider the inclusion of as a fixed factor to clarify its role in cognitive outcomes and ensure a more nuanced understanding of individual variability in response to cognitive tasks.

Regarding the physiological variables analyzed, the intermittent normobaric hypoxia induced a significant decrease in SaO_2_ and an increase in HR, with no relevant changes recorded in systolic or diastolic blood pressure. Previous studies have shown similar results (Foster et al., 2005; Zhang et al., 2014), suggesting that these changes could reflect the body’s acute compensatory mechanisms to maintain oxygen supply when its availability is reduced (Foster et al., 2005; Zhang et al., 2014). More specifically, the decrease in SaO_2_ could be the direct result of breathing air with a lower oxygen content, while the increase in HR could help maintain tissue oxygenation by increasing cardiac output (Foster et al., 2005; Zhang et al., 2014). For their part, systolic and diastolic blood pressure do not usually show significant changes during acute or short-term exposures to intermittent normobaric hypoxia (Foster et al., 2005; Zhang et al., 2014). Multiple studies in young and elderly adults, as well as in healthy and clinical populations, consistently report stable blood pressure values during episodes of hypoxia at FiO₂ levels ranging from 10 to 12%, even when SaO₂ decreases and HR increases (Foster et al., 2005; Zhang et al., 2014; Liu et al., 2020). This suggests that the cardiovascular system prioritizes heart rate adjustments over blood pressure modulation in response to acute hypoxic stress (Shi et al., 2017).

Based on the results of this study, the hypothesis can be accepted, since a single session of intermittent normobaric hypoxia might not impair executive functions in healthy young individuals regardless of sex. Given the absence of detrimental effects on executive functions finding in the current study, acute intermittent normobaric hypoxia protocol simulated altitude of 4,400 m above sea level with an oxygen saturation of 12% may be sure to use in different populations. This could have practical applications in both performance and clinical settings (Navarrete-Opazo and Mitchell, 2014). In sports contexts, understanding immediate effects may also have practical applications in contexts where acute IH exposure could be beneficial, such as short-term performance enhancement, pre-acclimatization before altitude exposure, or cognitive resilience training in occupational or military settings (Faiss et al., 2013). This kind of hypoxia protocols are frequently used in high-performance to achieve the physiological adaptations resulting from altitude training or military populations that operate under stressful or low-oxygen environments, where maintaining attention is critical (Post et al., 2023). In this context, considering the results of this research, hypoxia sessions can be carried out to achieve the desired physiological adaptations without any apparent acute risk of worsening the athlete’s cognitive response. This is very valuable information for training and competition planning, because tasks that require executive function can be carried out as normal after hypoxia sessions. In clinical settings, acute IH exposure may hold promise for individuals with attention deficits or age-related cognitive decline (Chen et al., 2023; Boulares et al., 2024; Zhang G. et al., 2024). Likewise, individuals undergoing cognitive rehabilitation or older adults at risk of cognitive decline might benefit from controlled exposure, provided it is applied within safe clinical parameters (Gonzalez-Rothi et al., 2015; Baillieul et al., 2017). Therefore, identifying the immediate effects of a single IH session in this clinical context is a relevant first step to ensure the safety and tolerability of IH protocols. If cognitive performance were to decline immediately after exposure, individuals might experience temporary impairments that could interfere with daily or occupational activities. Our findings indicate that this is not the case, suggesting that participants can safely resume normal activities following a session. Moreover, analyzing the acute response helps establish a baseline for future studies investigating cumulative or chronic effects of IH.

Despite the novelty of this research, this study has some limitations that should be acknowledged. Although a power analysis was conducted and the required sample size was met, the group sizes remain small and may be underpowered to detect subtle or moderate cognitive changes. Additionally, the study assessed only the acute effects of a single IH session. As such, the findings cannot be generalized to long-term or repeated exposures, and future research is needed to evaluate whether sustained protocols could produce different or more pronounced cognitive outcomes. Another limitation is the absence of a control group in which participants undergo simulated hypoxia, for example with mask use or simulator exposure, to analyze the placebo effect of IH exposure on physiological variables and cognitive task performance. Furthermore, the control group did not breathe normoxic air through a mask, but by breathing air naturally. Moreover, the potential for familiarity with the memory tests could have led to a learning effect across testing sessions, which may have influenced performance improvements in both EG and CG in Double Trouble Test. In addition, it was not possible to analyze the practice effect from the familiarization sessions, as the data from these sessions were not collected; however, the number of familiarization sessions was selected based on previous studies (Goldberg et al., 2015). Also, the cognitive tasks employed targeted specific executive functions like visuospatial working memory, response inhibition, and deductive reasoning, rather than general problem-solving, which constrains the generalizability of cognitive outcomes. All participants also had a similar educational level (higher education), which reduces the generalizability of the findings to broader populations with varying educational backgrounds. Finally, although sex-specific analyses were included due to their potential relevance, the small number of female and male participants per group strongly limits the interpretability of these findings, and they should be confirmed in larger samples. Future studies should consider larger and more diverse samples, repeated hypoxia exposures, and the inclusion of sham-controlled groups to strengthen the validity and applicability of results.

## 5 Conclusion

A single session of intermittent normobaric hypoxia did not impair executive function in healthy adults. The main contribution of this study lies in reporting that, under the tested conditions, acute intermittent normobaric hypoxia did not produce measurable changes in overall executive function in young adults.

## Data Availability

The raw data supporting the conclusions of this article will be made available by the authors, without undue reservation.

## References

[ref1] Albertus-Cámara I. Rochel-Vera C. Lomas-Albaladejo J.-L. Ferrer-López V. Martínez-González-Moro I. (2023). Ventilatory pattern influences tolerance to Normobaric hypoxia in healthy adults. Int. J. Environ. Res. Public Health 20:4935. doi: 10.3390/ijerph20064935, 36981844 PMC10049086

[ref2] Ando S. Komiyama T. Sudo M. Higaki Y. Ishida K. Costello J. T. . (2020). The interactive effects of acute exercise and hypoxia on cognitive performance: a narrative review. Scand. J. Med. Sci. Sports 30, 384–398. doi: 10.1111/sms.13573, 31605635

[ref3] Anguera J. A. Boccanfuso J. Rintoul J. L. Al-Hashimi O. Faraji F. Janowich J. . (2013). Video game training enhances cognitive control in older adults. Nature 501, 97–101. doi: 10.1038/nature12486, 24005416 PMC3983066

[ref4] Aragón-Vela J. Bejder J. Huertas R. Plaza-Diaz J. Nordsborg N. B. (2020). Does intermittent exposure to high altitude increase the risk of cardiovascular disease in workers? A systematic narrative review. BMJ Open 10:e041532. doi: 10.1136/bmjopen-2020-041532, 33444211 PMC7682469

[ref5] Baillieul S. Chacaroun S. Doutreleau S. Detante O. Pépin J. L. Verges S. (2017). Hypoxic conditioning and the central nervous system: a new therapeutic opportunity for brain and spinal cord injuries? Exp. Biol. Med. 242, 1198–1206. doi: 10.1177/1535370217712691, 28585890 PMC5478009

[ref6] Basovich S. N. (2010). The role of hypoxia in mental development and in the treatment of mental disorders: a review. Biosci. Trends 4, 288–296, 21248426

[ref8] Behrens M. (2022). New insights into the effects of acute intermittent hypoxia on neural plasticity in the human motor system. Exp. Physiol. 107, 560–561. doi: 10.1113/EP090462, 35462446

[ref9] Boulares A. Pichon A. Faucher C. Bragazzi N. L. Dupuy O. (2024). Effects of intermittent hypoxia protocols on cognitive performance and brain health in older adults across cognitive states: a systematic literature review. J Alzheimer's Dis 101, 13–30. doi: 10.3233/JAD-240711, 39093075

[ref10] Chen X. Zhang J. Lin Y. Li Y. Wang H. Wang Z. . (2023). Mechanism, prevention and treatment of cognitive impairment caused by high altitude exposure. Front. Physiol. 14:1191058. doi: 10.3389/fphys.2023.1191058 37731540 PMC10507266

[ref11] Chen L. Zheng Z. Liang J. Lin Y. Miao Q. (2024). Understanding gender differences in reasoning and specific paradigm using meta-analysis of neuroimaging. Front. Behav. Neurosci. 18:1457663. doi: 10.3389/fnbeh.2024.1457663, 39839537 PMC11747635

[ref12] Chroboczek M. Kostrzewa M. Micielska K. Grzywacz T. Laskowski R. (2021). Effect of acute Normobaric hypoxia exposure on executive functions among Young physically active males. J. Clin. Med. 10:1560. doi: 10.3390/jcm10081560, 33917691 PMC8068023

[ref13] Chroboczek M. Kujach S. Łuszczyk M. Grzywacz T. Soya H. Laskowski R. (2022). Acute Normobaric hypoxia lowers executive functions among Young men despite increase of BDNF concentration. Int. J. Environ. Res. Public Health 19:802. doi: 10.3390/ijerph191710802 36078520 PMC9518314

[ref14] Coppel J. Hennis P. Gilbert-Kawai E. Grocott M. P. (2015). The physiological effects of hypobaric hypoxia versus normobaric hypoxia: a systematic review of crossover trials. Extrem Physiol Med 4:2. doi: 10.1186/s13728-014-0021-6 25722851 PMC4342204

[ref15] Dale E. A. Ben Mabrouk F. Mitchell G. S. (2014). Unexpected benefits of intermittent hypoxia: enhanced respiratory and nonrespiratory motor function. Physiology 29, 39–48. doi: 10.1152/physiol.00012.2013, 24382870 PMC4073945

[ref16] Davranche K. Casini L. Arnal P. J. Rupp T. Perrey S. Verges S. (2016). Cognitive functions and cerebral oxygenation changes during acute and prolonged hypoxic exposure. Physiol. Behav. 164, 189–197. doi: 10.1016/j.physbeh.2016.06.001, 27262217

[ref17] Diamond A. (2013). Executive functions. Annu. Rev. Psychol. 64, 135–168. doi: 10.1146/annurev-psych-113011-143750, 23020641 PMC4084861

[ref18] Donnan K. Williams E. L. Stanger N. (2021). The effects of heat exposure during intermittent exercise on physical and cognitive performance among team Sport athletes. Percept. Mot. Skills 128, 439–466. doi: 10.1177/0031512520966522, 33076764 PMC7859587

[ref19] Faiss R. Girard O. Millet G. P. (2013). Advancing hypoxic training in team sports: from intermittent hypoxic training to repeated sprint training in hypoxia. Br. J. Sports Med. 47, i45–i50. doi: 10.1136/bjsports-2013-092741, 24282207 PMC3903143

[ref20] Foster G. E. McKenzie D. C. Milsom W. K. Sheel A. W. (2005). Effects of two protocols of intermittent hypoxia on human ventilatory, cardiovascular and cerebral responses to hypoxia. J. Physiol. 567, 689–699. doi: 10.1113/jphysiol.2005.091462, 15975977 PMC1474187

[ref21] Galea L. A. M. Frick K. M. Hampson E. Sohrabji F. Choleris E. (2017). Why estrogens matter for behavior and brain health. Neurosci. Biobehav. Rev. 76, 363–379. doi: 10.1016/j.neubiorev.2016.03.024, 27039345 PMC5045786

[ref22] Gangwar A. Pooja Sharma M. Singh K. Patyal A. Bhaumik G. . (2019). Intermittent normobaric hypoxia facilitates high altitude acclimatization by curtailing hypoxia-induced inflammation and dyslipidemia. Pflugers Arch. 471, 949–959. doi: 10.1007/s00424-019-02273-4, 30980137

[ref23] Glazachev O. Kopylov P. Susta D. Dudnik E. Zagaynaya E. (2017). Adaptations following an intermittent hypoxia-hyperoxia training in coronary artery disease patients: a controlled study. Clin. Cardiol. 40, 370–376. doi: 10.1002/clc.22670, 28323322 PMC6490434

[ref24] Goldberg T. E. Harvey P. D. Wesnes K. A. Snyder P. J. Schneider L. S. (2015). Practice effects due to serial cognitive assessment: implications for preclinical Alzheimer's disease randomized controlled trials. Alzheimers Dement. 1, 103–111. doi: 10.1016/j.dadm.2014.11.003, 27239497 PMC4876902

[ref25] Gonzalez-Rothi E. J. Lee K.-Z. Dale E. A. Reier P. J. Mitchell G. S. Fuller D. D. (2015). Intermittent hypoxia and neurorehabilitation. J. Appl. Physiol. 1985, 1455–1465. doi: 10.1152/japplphysiol.00235.2015 PMC4683349 25997947

[ref26] Grenell A. Carlson S. M. (2021). Individual differences in executive function and learning: the role of knowledge type and conflict with prior knowledge. J. Exp. Child Psychol. 206:105079. doi: 10.1016/j.jecp.2020.105079, 33610883 PMC8900212

[ref27] Guo M. Yu W. Sun Y. Wang L. Zhou H. Zhang Y. (2023). Effects of increasing indoor negative air ions on cognitive performance and health of high pure CO2Level-exposed college students. Indoor Air 2023:8339. doi: 10.1155/2023/8298339

[ref28] Hayes H. B. Jayaraman A. Herrmann M. Mitchell G. S. Rymer W. Z. Trumbower R. D. (2014). Daily intermittent hypoxia enhances walking after chronic spinal cord injury: a randomized trial. Neurology 82, 104–113. doi: 10.1212/01.WNL.0000437416.34298.43, 24285617 PMC3897437

[ref29] Heinemann K. (2003). Introducción a la metodología de la investigación empírica en las ciencias del deporte. Badalona, Spain: Paidotribo.

[ref30] Honarmand K. Malik S. Wild C. Gonzalez-Lara L. E. McIntyre C. W. Owen A. M. . (2019). Feasibility of a web-based neurocognitive battery for assessing cognitive function in critical illness survivors. PLoS One 14:e0215203. doi: 10.1371/journal.pone.0215203, 30978210 PMC6461230

[ref31] Hopkins W. G. Marshall S. W. Batterham A. M. Hanin J. (2009). Progressive statistics for studies in sports medicine and exercise science. Med. Sci. Sports Exerc. 41, 3–13. doi: 10.1249/MSS.0b013e31818cb278, 19092709

[ref32] Hosseini M. Borhani-Haghighi A. Petramfar P. Foroughi A. A. Ostovan V. R. Nami M. (2023). Evaluating cognitive impairment in the early stages of Parkinson's disease using the Cambridge brain sciences-cognitive platform. Clin. Neurol. Neurosurg. 232:107866. doi: 10.1016/j.clineuro.2023.107866, 37413872

[ref33] Hurt R. T. Ganesh R. Schroeder D. R. Hanson J. L. Fokken S. C. Overgaard J. D. . (2025). Using a wearable brain activity sensing device in the treatment of long COVID symptoms in an open-label clinical trial. J. Prim. Care Community Health 16:21501319251325639. doi: 10.1177/21501319251325639 40071827 PMC11905036

[ref34] Hynes S. M. Fish J. Manly T. (2014). Intensive working memory training: a single case experimental design in a patient following hypoxic brain damage. Brain Inj. 28, 1766–1775. doi: 10.3109/02699052.2014.954622, 25207877

[ref35] Issa A. N. Herman N. M. Wentz R. J. Taylor B. J. Summerfield D. C. Johnson B. D. (2016). Association of cognitive performance with time at altitude, sleep quality, and acute mountain sickness symptoms. Wilderness Environ. Med. 27, 371–378. doi: 10.1016/j.wem.2016.04.008, 27460198

[ref36] Janssen Daalen J. M. Meinders M. J. Giardina F. Roes K. C. B. Stunnenberg B. C. Mathur S. . (2022). Multiple N-of-1 trials to investigate hypoxia therapy in Parkinson’s disease: study rationale and protocol. BMC Neurol. 22:262. doi: 10.1186/s12883-022-02770-7 35836147 PMC9281145

[ref37] Kadam P. Bhalerao S. (2010). Sample size calculation. Int J Ayurveda Res 1, 55–57. doi: 10.4103/0974-7788.59946, 20532100 PMC2876926

[ref38] Karayigit R. Eser M. C. Sahin F. N. Sari C. Sanchez-Gomez A. Dominguez R. . (2022a). The acute effects of Normobaric hypoxia on strength, muscular endurance and cognitive function: influence of dose and sex. Biology 11:309. doi: 10.3390/biology11020309 35205175 PMC8869765

[ref39] Karayigit R. Ramirez-Campillo R. Yasli B. C. Gabrys T. Benesova D. Esen O. (2022b). High dose of acute Normobaric hypoxia does not adversely affect Sprint interval training, cognitive performance and heart rate variability in males and females. Biology 11:1463. doi: 10.3390/biology11101463 36290367 PMC9598265

[ref40] Kazali E. (2025). Executive functions in inductive and deductive reasoning. J. Exp. Child Psychol. 252:106144. doi: 10.1016/j.jecp.2024.106144, 39673822

[ref42] Koehler U. Hildebrandt O. Krönig J. Grimm W. Otto J. Hildebrandt W. . (2018). Chronic hypoxia and cardiovascular risk: clinical significance of different forms of hypoxia. Herz 43, 291–297. doi: 10.1007/s00059-017-4570-5, 28474128

[ref43] Komiyama T. Sudo M. Higaki Y. Kiyonaga A. Tanaka H. Ando S. (2015). Does moderate hypoxia alter working memory and executive function during prolonged exercise? Physiol. Behav. 139, 290–296. doi: 10.1016/j.physbeh.2014.11.057, 25460539

[ref44] Lei O. K. Kong Z. Loprinzi P. D. Shi Q. Sun S. Zou L. . (2019). Severe hypoxia does not offset the benefits of exercise on cognitive function in sedentary Young women. Int. J. Environ. Res. Public Health 16:1003. doi: 10.3390/ijerph16061003, 30897697 PMC6466299

[ref45] Lim C.-Y. In J. (2019). Randomization in clinical studies. Korean J. Anesthesiol. 72, 221–232. doi: 10.4097/kja.19049, 30929415 PMC6547231

[ref46] Limmer M. Platen P. (2018). The influence of hypoxia and prolonged exercise on attentional performance at high and extreme altitudes: a pilot study. PLoS One 13:e0205285. doi: 10.1371/journal.pone.0205285, 30281651 PMC6169942

[ref47] Liu X. Chen X. Kline G. Ross S. E. Hall J. R. Ding Y. . (2020). Reduced cerebrovascular and cardioventilatory responses to intermittent hypoxia in elderly. Respir. Physiol. Neurobiol. 271:103306. doi: 10.1016/j.resp.2019.103306, 31557538

[ref48] Manukhina E. B. Downey H. F. Shi X. Mallet R. T. (2016). Intermittent hypoxia training protects cerebrovascular function in Alzheimer’s disease. Exp. Biol. Med. 241, 1351–1363. doi: 10.1177/1535370216649060, 27190276 PMC4950272

[ref49] McMorris T. Hale B. J. Barwood M. Costello J. Corbett J. (2017). Effect of acute hypoxia on cognition: a systematic review and meta-regression analysis. Neurosci. Biobehav. Rev. 74, 225–232. doi: 10.1016/j.neubiorev.2017.01.019, 28111267

[ref50] Medina-Inojosa J. R. Ibarra M. A. G. Medina-Inojosa B. J. Supervia M. Jenkins S. Johnson L. . (2024). Effect of active workstations on neurocognitive performance and typing skills: a randomized clinical trial. J. Am. Heart Assoc. 13, 1–10. doi: 10.1161/JAHA.123.031228 PMC11262529 38572691

[ref51] Metzler-Baddeley C. Caeyenberghs K. Foley S. Jones D. K. (2016). Task complexity and location specific changes of cortical thickness in executive and salience networks after working memory training. NeuroImage 130, 48–62. doi: 10.1016/j.neuroimage.2016.01.007, 26806288 PMC4819728

[ref52] Moser P. (2019). Out of control? Managing baseline variability in experimental studies with control groups, (Cham, Switzerland: Springer) 101–117. 10.1007/164_2019_280 31595416

[ref53] Navarrete-Opazo A. Mitchell G. S. (2014). Therapeutic potential of intermittent hypoxia: a matter of dose. Am. J. Physiol. Regul. Integr. Comp. Physiol. 307, R1181–R1197. doi: 10.1152/ajpregu.00208.2014, 25231353 PMC4315448

[ref54] Neuhaus C. Hinkelbein J. (2014). Cognitive responses to hypobaric hypoxia: implications for aviation training. Psychol. Res. Behav. Manag. 7, 297–302. doi: 10.2147/PRBM.S51844, 25419162 PMC4234165

[ref55] Nichols E. S. Erez J. Stojanoski B. Lyons K. M. Witt S. T. Mace C. A. . (2021a). Longitudinal white matter changes associated with cognitive training. Hum. Brain Mapp. 42, 4722–4739. doi: 10.1002/hbm.25580 34268814 PMC8410562

[ref56] Nichols E. S. Wild C. J. Owen A. M. Soddu A. (2021b). Cognition across the lifespan: investigating age, sex, and other sociodemographic influences. Behav. Sci. 11:51. doi: 10.3390/bs11040051 33924660 PMC8070049

[ref57] Ochi G. Yamada Y. Hyodo K. Suwabe K. Fukuie T. Byun K. . (2018). Neural basis for reduced executive performance with hypoxic exercise. NeuroImage 171, 75–83. doi: 10.1016/j.neuroimage.2017.12.091, 29305162

[ref58] Owen A. M. Hampshire A. Grahn J. A. Stenton R. Dajani S. Burns A. S. . (2010). Putting brain training to the test. Nature 465, 775–778. doi: 10.1038/nature09042, 20407435 PMC2884087

[ref59] Patrick Z. Sepko J. W. Loprinzi P. D. (2023). The effects of acute hypoxia exposure on cognitive function. Med. Sci. Sports Exerc. 55:606. doi: 10.1249/01.mss.0000985464.98507.60

[ref60] Peltonen G. L. Harrell J. W. Aleckson B. P. LaPlante K. M. Crain M. K. Schrage W. G. (2016). Cerebral blood flow regulation in women across menstrual phase: differential contribution of cyclooxygenase to basal, hypoxic, and hypercapnic vascular tone. Am. J. Physiol. Regul. Integr. Comp. Physiol. 311, R222–R231. doi: 10.1152/ajpregu.00106.2016, 27225949 PMC5008661

[ref61] Pithon M. M. (2013). Importance of the control group in scientific research. Dental Press J. Orthod. 18, 13–14. doi: 10.1590/S2176-94512013000600003, 24498644

[ref62] Post T. E. Heijn L. G. Jordan J. van Gerven J. M. A. (2023). Sensitivity of cognitive function tests to acute hypoxia in healthy subjects: a systematic literature review. Front. Physiol. 14:1244279. doi: 10.3389/fphys.2023.1244279 37885803 PMC10598721

[ref63] Prabhakar N. R. Peng Y.-J. Nanduri J. (2022). Adaptive cardiorespiratory changes to chronic continuous and intermittent hypoxia. Handb. Clin. Neurol. 188, 103–123. doi: 10.1016/B978-0-323-91534-2.00009-6, 35965023 PMC9906984

[ref64] Pun M. Guadagni V. Bettauer K. M. Drogos L. L. Aitken J. Hartmann S. E. . (2018). Effects on cognitive functioning of acute, subacute and repeated exposures to high altitude. Front. Physiol. 9, 1–15. doi: 10.3389/fphys.2018.01131 30246787 PMC6111975

[ref65] Pun M. Guadagni V. Drogos L. L. Pon C. Hartmann S. E. Furian M. . (2019). Cognitive effects of repeated acute exposure to very high altitude among altitude-experienced workers at 5050 m. High Alt. Med. Biol. 20, 361–374. doi: 10.1089/ham.2019.0012, 31651199

[ref66] Raberin A. Burtscher J. Burtscher M. Millet G. P. (2023). Hypoxia and the aging cardiovascular system. Aging Dis. 14, 2051–2070. doi: 10.14336/AD.2023.0424, 37199587 PMC10676797

[ref67] Raberin A. Burtscher J. Citherlet T. Manferdelli G. Krumm B. Bourdillon N. . (2024). Women at altitude: sex-related physiological responses to exercise in hypoxia. Sports Med. 54, 271–287. doi: 10.1007/s40279-023-01954-6, 37902936 PMC10933174

[ref68] Ramírez-de la Cruz M. Ortiz-Sánchez D. Bravo-Sánchez A. Portillo J. Esteban-García P. Abián-Vicén J. (2025). Effects of different exposures to normobaric hypoxia on cognitive performance in healthy young adults.: Normobaric hypoxia and cognitive performance. Physiol. Behav. 288:4747. doi: 10.1016/j.physbeh.2024.114747 39547435

[ref69] Riganello F. Pearce A. Lyons K. M. Owen A. M. Soddu A. Stojanoski B. (2023). Differential effects of prolonged aerobic and resistance exercise on cognitive functioning in sedentary young adults. Europe PMC:PPR641647. doi: 10.1101/2023.04.03.535439

[ref70] Schega L. Peter B. Törpel A. Mutschler H. Isermann B. Hamacher D. (2013). Effects of intermittent hypoxia on cognitive performance and quality of life in elderly adults: a pilot study. Gerontology 59, 316–323. doi: 10.1159/000350927, 23652274

[ref71] Schulz K. F. Altman D. G. Moher D. (2010). CONSORT 2010 statement: updated guidelines for reporting parallel group randomised trials. BMJ 340:c332. doi: 10.1136/bmj.c332, 20332509 PMC2844940

[ref72] Shi X. Liu X. Xu D. Ross S. Hall J. O’Bryant S. (2017). Normobaric intermittent hypoxia increases middle cerebral arterial blood flow velocity with no systemic hypertension. Med. Sci. Sports Exerc. 49:61. doi: 10.1249/01.mss.0000516992.99870.3c

[ref73] Stark R. Wolf O. T. Tabbert K. Kagerer S. Zimmermann M. Kirsch P. . (2006). Influence of the stress hormone cortisol on fear conditioning in humans: evidence for sex differences in the response of the prefrontal cortex. NeuroImage 32, 1290–1298. doi: 10.1016/j.neuroimage.2006.05.046, 16839780

[ref74] Sternin A. Burns A. Owen A. M. (2019). Thirty-five years of computerized cognitive assessment of aging-where are we now? Diagnostics 9:114. doi: 10.3390/diagnostics9030114, 31489940 PMC6787729

[ref75] Sujanthan S. Puveendrakumaran P. Dainty K. N. Barense M. Lanctot K. L. Owen A. M. . (2025). Feasibility of telephone and computerized cognitive testing as a secondary outcome in an acute stroke clinical trial: a mixed methods sub-study of the AcT trial. Eur. Stroke J. 10, 968–977. doi: 10.1177/23969873251323171, 40071564 PMC11907497

[ref76] Timon R. Martinez-Guardado I. Brocherie F. (2023). Effects of intermittent Normobaric hypoxia on health-related outcomes in healthy older adults: a systematic review. Sports Med Open 9:19. doi: 10.1186/s40798-023-00560-0 36843041 PMC9968673

[ref77] Törpel A. Peter B. Hamacher D. Schega L. (2019). Dose-response relationship of intermittent normobaric hypoxia to stimulate erythropoietin in the context of health promotion in young and old people. Eur. J. Appl. Physiol. 119, 1065–1074. doi: 10.1007/s00421-019-04096-8, 30756167

[ref78] Törpel A. Peter B. Schega L. (2020). Effect of resistance training under Normobaric hypoxia on physical performance, Hematological parameters, and body composition in Young and older people. Front. Physiol. 11:335. doi: 10.3389/fphys.2020.00335 32411007 PMC7198789

[ref79] Turner C. E. Barker-Collo S. L. Connell C. J. W. Gant N. (2015). Acute hypoxic gas breathing severely impairs cognition and task learning in humans. Physiol. Behav. 142, 104–110. doi: 10.1016/j.physbeh.2015.02.006, 25660759

[ref80] Twomey R. Wrightson J. Fletcher H. Avraam S. Ross E. Dekerle J. (2017). Exercise-induced fatigue in severe hypoxia after an intermittent hypoxic protocol. Med. Sci. Sports Exerc. 49, 2422–2432. doi: 10.1249/MSS.0000000000001371, 28708702

[ref81] Uchida K. Baker S. E. Wiggins C. C. Senefeld J. W. Shepherd J. R. A. Trenerry M. R. . (2020). A novel method to measure transient impairments in cognitive function during acute bouts of hypoxia. Aerosp Med Hum Perform 91, 839–844. doi: 10.3357/AMHP.5665.2020, 33334403

[ref82] Vento K. A. Borden C. K. Blacker K. J. (2022). Sex comparisons in physiological and cognitive performance during hypoxic challenge. Front. Physiol. 13:1062397. doi: 10.3389/fphys.2022.1062397 36505049 PMC9727089

[ref83] Virués-Ortega J. Buela-Casal G. Garrido E. Alcázar B. (2004). Neuropsychological functioning associated with high-altitude exposure. Neuropsychol. Rev. 14, 197–224. doi: 10.1007/s11065-004-8159-4, 15796116

[ref84] Wang H. Shi X. Schenck H. Hall J. R. Ross S. E. Kline G. P. . (2020). Intermittent hypoxia training for treating mild cognitive impairment: a pilot study. Am. J. Alzheimers Dis. Other Dement. 35, 1–10. doi: 10.1177/1533317519896725 PMC10624018 31902230

[ref85] Wild C. Norton L. Menon D. Ripsman D. Swartz R. Owen A. (2022). Seeing through brain fog: disentangling the cognitive, physical, and mental health sequalae of COVID-19. Cell Rep Med 3, 10050. doi: 10.21203/rs.3.rs-373663/v1 PMC9448696 36103880

[ref86] Wild C. J. Norton L. Menon D. K. Ripsman D. A. Swartz R. H. Owen A. M. (2022). Disentangling the cognitive, physical, and mental health sequelae of COVID-19. Cell Rep Med 3:100750. doi: 10.1016/j.xcrm.2022.100750, 36103880 PMC9448696

[ref87] Williams T. B. Corbett J. McMorris T. Young J. S. Dicks M. Ando S. . (2019). Cognitive performance is associated with cerebral oxygenation and peripheral oxygen saturation, but not plasma catecholamines, during graded normobaric hypoxia. Exp. Physiol. 104, 1384–1397. doi: 10.1113/EP087647, 31192502

[ref88] Xing T. Pilowsky P. M. Fong A. Y. (2014). Mechanism of sympathetic activation and blood pressure elevation in humans and animals following acute intermittent hypoxia. Prog. Brain Res. 209, 131–146. doi: 10.1016/B978-0-444-63274-6.00007-2 24746046

[ref89] Xu L. Wu Y. Zhao T. Liu S.-H. Zhu L.-L. Fan M. . (2014). Effect of high altitude hypoxia on cognitive flexibility. Zhongguo Ying Yong Sheng Li Xue Za Zhi 30:118. 25016857

[ref90] Yuan L. Kong F. Luo Y. Zeng S. Lan J. You X. (2019). Gender differences in large-scale and small-scale spatial ability: a systematic review based on Behavioral and neuroimaging research. Front. Behav. Neurosci. 13:128. doi: 10.3389/fnbeh.2019.00128 31275121 PMC6591491

[ref91] Yuan H. Liu J. Gu Y. Ji X. Nan G. (2022). Intermittent hypoxia conditioning as a potential prevention and treatment strategy for ischemic stroke: current evidence and future directions. Front. Neurosci. 16:1067411. doi: 10.3389/fnins.2022.1067411 36507357 PMC9732261

[ref92] Zhang P. Downey H. F. Chen S. Shi X. (2014). Two-week normobaric intermittent hypoxia exposures enhance oxyhemoglobin equilibrium and cardiac responses during hypoxemia. Am. J. Phys. Regul. Integr. Comp. Phys. 307, R721–R730. doi: 10.1152/ajpregu.00191.2014, 25056104

[ref93] Zhang Q. Wang Q. Jin F. Huang D. Ji X. Wang Y. (2024). Intermittent hypoxia training improves cerebral blood flow without cognitive impairment. Ann. Clin. Transl. Neurol., 12:86–96. doi: 10.1002/acn3.52248 39543930 PMC11752099

[ref94] Zhang G. Yang G. Zhou Y. Cao Z. Yin M. Ma L. . (2024). Intermittent hypoxia training effectively protects against cognitive decline caused by acute hypoxia exposure. Pflugers Arch. 476, 197–210. doi: 10.1007/s00424-023-02885-x, 37994929

[ref95] Zhang Q. Zhao W. Li S. Ding Y. Wang Y. Ji X. (2023). Intermittent hypoxia conditioning: a potential multi-organ protective therapeutic strategy. Int. J. Med. Sci. 20, 1551–1561. doi: 10.7150/ijms.86622, 37859700 PMC10583178

